# Cystic echinococcosis in donkeys in eastern Africa

**DOI:** 10.1017/S0031182023000173

**Published:** 2023-04

**Authors:** Erastus Mulinge, Eberhard Zeyhle, Cecilia Mbae, Lucy Gitau, Timothy Kaburu, Japhet Magambo, Ute Mackenstedt, Thomas Romig, Peter Kern, Marion Wassermann

**Affiliations:** 1 Kenya Medical Research Institute, Nairobi, Kenya; 2 Meru University of Science and Technology, Meru, Kenya; 3 Parasitology Unit, University of Hohenheim, Stuttgart, Germany; 4 Department of Medicine III, University Hospital Ulm, Ulm, Germany

**Keywords:** Cystic echinococcosis, donkeys, eastern Africa, *Echinococcus equinus*, *Echinococcus granulosus* sensu lato

## Abstract

Cystic echinococcosis (CE) is endemic in humans and domestic animals in eastern Africa. All the species of the *Echinococcus granulosus* sensu lato complex have been reported in this region except for *E. equinus*, possibly due to the small number of studies involving equids. This study reports the frequency of different *Echinococcus* species in donkeys from eastern Africa. A total of 5961 donkeys were examined during meat inspection in 3 slaughterhouses in Kenya. Identification of *Echinococcus* spp. was achieved through polymerase chain reaction-restriction fragment-length polymorphism and sequencing of the mitochondrial nicotinamide adenine dinucleotide (NADH) dehydrogenase subunit 1 gene. The prevalence of CE was 5.7% (337/5961). The 263 genotyped cysts belonged to *E. equinus* (*n* = 163), *E. granulosus* sensu stricto (*n* = 70), *E. canadensis* (G6/7) (*n* = 26) and *E. ortleppi* (*n* = 4). One donkey harboured a metacestode of *Spirometra theileri*. All *E. equinus* cases, except 2, originated from southern Ethiopia, whereas the other species were more evenly distributed across the study area. Most of the cysts belonging to *E. equinus* were fertile (111/163), while those of the other species were non-fertile. This is the first report of *Echinococcus* spp. in donkeys from sub-Saharan Africa and the first confirmation of *E. equinus* in East Africa. The frequent fertility of *E. equinus* cysts in donkeys affirms their suitability as intermediate hosts of this species, while low frequency and cyst fertility suggest a marginal role of donkeys in the transmission of *E. granulosus* s. s., *E. canadensis* (G6/7) and *E. ortleppi*.

## Graphical abstract

**Figure:**
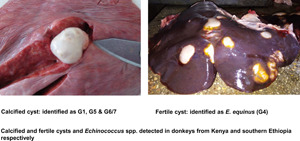

## Introduction

Cystic echinococcosis (CE) is a zoonotic disease caused by metacestodes of the cestode *Echinococcus granulosus* sensu lato (s. l.). The World Health Organization (WHO) has listed echinococcosis among 20 neglected diseases targeted for control or elimination by 2050 (WHO, 2012). Although CE has a worldwide distribution, it has major public health and economic impact in areas of extensive livestock keeping (Deplazes *et al*., 2017). The lifecycles of the various species within *E. granulosus* s. l. involve mainly canids as definitive hosts, and a range of herbivorous or omnivorous intermediate hosts in which the metacestode develops (Thompson, 2017).

The *E. granulosus* s. l. complex includes at least 5 cryptic species and some distinct genotypes (G), namely *E*. *granulosus* sensu stricto (s. s.) (G1, G3, G*Omo*), *E*. *equinus* (G4), *E*. *ortleppi* (G5), *E*. *canadensis* (G6–8, G10) and *E*. *felidis* (Nakao *et al*., 2013*a*, 2013*b*; Wassermann *et al*., 2016; Vuitton *et al*., 2020). Epidemiological studies in eastern Africa have reported all these taxa except for *E. canadensis* G8 and G10 (which are wildlife parasites from the northern Holarctic) and *E. equinus*. The latter mainly infects intermediate hosts of the horse family (Equidae), is rarely zoonotic and occurs around the globe mainly in domestic lifecycles involving dogs and horses or donkeys (Romig *et al*., 2017).

In Africa, *E. equinus* is known to infect donkeys north of the Sahara at considerable frequency (Azlaf and Dakkak, 2006; Haridy *et al*., 2008; Taha, 2012; Aboelhadid *et al*., 2013; Boufana *et al*., 2014; Lahmar *et al*., 2014; Mahdy *et al*., 2014*a*, 2014*b*; Barghash *et al*., 2017; Desouky *et al*., 2017), and in southern Africa, it has been reported from horses, zebras and rhinos (Kumaratilake *et al*., 1986; Wassermann *et al*., 2015; Romig *et al*., 2017; Zaffarano *et al*., 2021). However, there are no records from the west, central and east of the continent. At least for eastern Africa, the absence of *E. equinus* can be explained by sampling bias. While numerous studies in the last decade contributed to our understanding of CE epidemiology, mainly in Kenya, by screening livestock, wildlife and humans (Kagendo *et al*., 2014; Mbaya *et al*., 2014; Addy *et al*., 2017*a*; Romig *et al*., 2017; Mulinge *et al*., 2018; Odongo *et al*., 2018; Kere *et al*., 2019; Nungari *et al*., 2019; Omondi *et al*., 2020), none of these surveys involved donkeys, horses or zebras. This was mainly due to the absence of slaughter facilities for donkeys, as they were not slaughtered for human consumption even though the government of Kenya had gazetted donkeys as food animals in 1999 (Legal Act Notice No146, 1999). Only recently, several export slaughterhouses for donkeys were licensed to operate in Kenya, which provided a research opportunity to close the gap of knowledge on the presence, frequency and causative species of CE in donkeys and to determine their role in the transmission cycles.

## Materials and methods

### Study sites

**Fig. 1.**
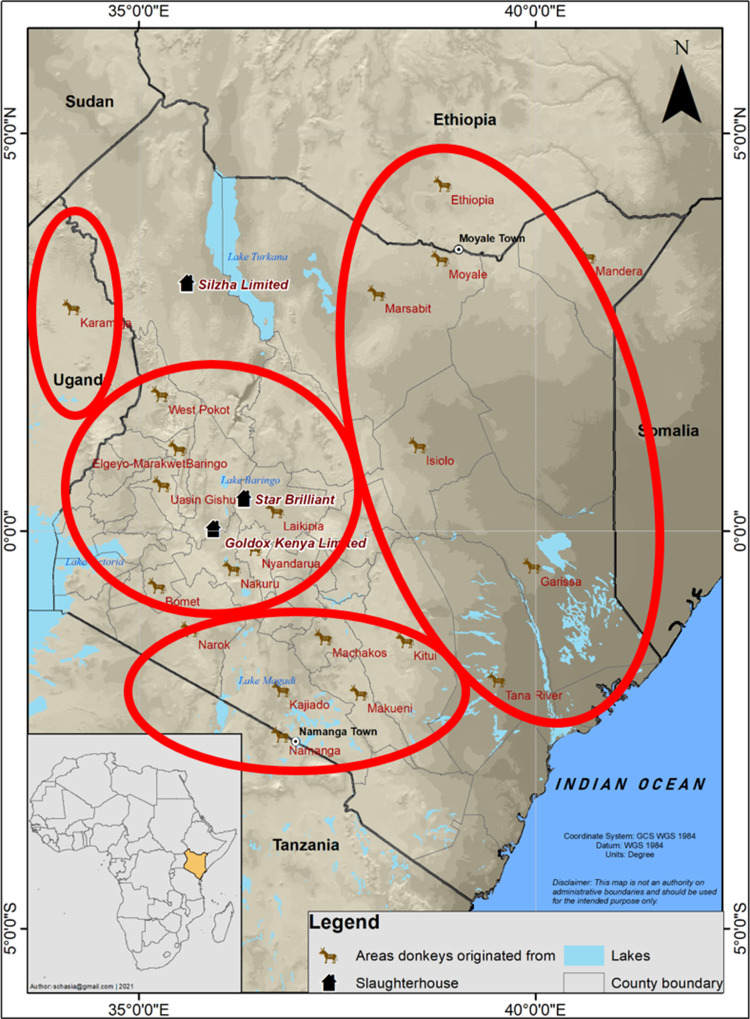
A map of eastern Africa showing the location of the 3 slaughterhouse, the origin of the donkeys in Kenya and the neighbouring countries, and the 4 sub-regions listed in Table 2.

The study was done in 3 private-owned slaughterhouses, 1 in Turkana County (Lodwar: Silzha Ltd.) and 2 in central Kenya (Naivasha: Star Brilliant Ltd. and Mogotio: Goldox Kenya Ltd.) (Fig. 1). The origin of the donkeys was identified to county level in Kenya; donkeys from Uganda were from the Karamoja area, donkeys from Tanzania were from the Maasai area in northern Tanzania, and donkeys from Ethiopia entering Kenya through the border town of Moyale originated from the adjacent Borena zone of southern Ethiopia (Fig. 1).

### Collection of CE cysts and microscopic examination

The study was done at 2 intervals; the first between January and September 2017 which involved all the 3 slaughterhouses (3197 donkeys). The second phase was carried out only in Naivasha slaughterhouse between February and August 2019 (2764 donkeys). Carcasses were examined for cysts by palpation and subsequent incision of the major organs. The cysts/lesions were excised *in toto*, cleaned and stored individually in 70% ethanol. The cysts were examined microscopically for the presence of protoscoleces, and with sterile cysts (not containing protoscoleces), the integrity of the germinal layer was visually assessed to discriminate between viable and degenerated cysts.

### DNA extraction

Cyst material or a single protoscolex were lysed in 0.02 M NaOH at 99°C for 10 min (Nakao *et al*., 2003). The lysate was used as template immediately following polymerase chain reaction (PCR). Cyst samples that failed to yield a PCR product following the lysis procedure were subjected to DNA extraction using DNeasy Blood and Tissue kit (Qiagen, Hilden, Germany) according to the manufacturer's instructions.

### Polymerase chain reaction-restriction fragment-length polymorphism, PCR purification and sequencing

Two nested PCR assays based on NADH dehydrogenase subunit 1 gene (*nad1*) were used for genotyping of cysts depending on their conditions. The first PCR targeted the entire *nad1* gene (894 bp) and was carried out according to Hüttner *et al*. (2008). The second PCR amplified part of the *nad1* gene (550–552 bp) as described by Mulinge *et al*. (2018) and was carried out on samples that failed to yield an amplicon with the first PCR. In both PCR assays, the reaction mixture contained 2 *μ*L of the DNA, 1 × DreamTaq Green Buffer [20 mm Tris-HCl (pH 8.0), 1 mm Dithiothreitol (DTT), 0.1 mm Ethylenediamine tetraacetic acid (EDTA), 100 mm KCl, 0.5% (v/v) Nonidet P40, 0.5% (v/v) Tween 20] (Thermo Scientific, Waltham, MA, USA), 0.2 mm deoxynucleotide triphosphate (New England Biolabs, Ipswich, MA, USA), 0.25 *μ*m of forward and reverse primers each, 2 mm MgCl_2_ and 0.625 units of DreamTaq Green DNA Polymerase (Thermo Scientific) in 25 *μ*L final volume. The PCR cycling conditions were 5 min for initial denaturation at 94°C, 40 cycles of 94°C for 30 s, 55°C for 30 s and 72°C for 60 s, and a final extension at 72°C for 5 min (Hüttner *et al*., 2008).

The *nad1* PCR products were digested using the restriction enzyme, *Hph*I (New England Biolabs), either the entire or partial *nad*1 gene (Hüttner *et al*., 2009; Mulinge *et al*., 2018). The total reaction mixture was 20 *μ*L including 7.5 *μ*L nuclease-free water, 2.0 *μ*L of 10 × CutSmart buffer, 0.5 *μ*L *Hph*I (5 units) and 10 *μ*L PCR product. The restriction digests were incubated overnight at 37°C and separated on 3% agarose gel alongside positive controls for *E. granulosus* s. s., *E. equinus*, *E. ortleppi*, *E. canadensis* (G6/7) and *E. felidis* (Hüttner *et al*., 2009). In addition, for confirmation purposes and in case of ambiguous banding patterns, PCR products were purified using QIAquick PCR purification kit (Qiagen) following the manufacturer's guidelines. The purified amplicons were sent to Macrogen Europe BV (Amsterdam, the Netherlands) for sequencing using the nested reverse primer. The sequences were identified by comparing with those available in the National Centre for Biotechnology Information database (NCBI) using the basic local alignment search tool (http://www.ncbi.nlm.nih.gov/BLAST/) (Altschul *et al*., 1997).

## Results

### Cyst condition and organ location of *Echinococcus* spp.

Out of 338 donkeys, 528 cystic structures were collected. Of these, 263 were characterized as *Echinococcus* spp. and 1 as a metacestode of *Spirometra theileri*. The remaining cysts either did not yield sequences or banding patterns of sufficient quality, or were excluded from molecular examination due to advanced degeneration with low probability of amplification success. Four *E. granulosus* s. l. species were detected: *E. equinus* (*n* = 163), *E. granulosus* s. s. (*n* = 70), *E. canadensis* (G6/7) (*n* = 26) and *E. ortleppi* (*n* = 4) (Tables 1 and 2). Details of representative sequences obtained in this study are available on GenBank under accession numbers OK489943–OK489955 (Table 3). Details of sequences from GenBank that were identical to those reported in this study are shown in Table 3. Fertility of *E. equinus* cysts was high (111/163), but low for the other species: 7/70 for *E. granulosus* s. s., 1/4 for *E. ortleppi* and 3/26 for *E. canadensis* (G6/7). The most frequently infected organs were the liver for *E. equinus* (115/163) and *E. canadensis* (G6/7) (15/26) and the lungs for *E. granulosus* s. s. (43/70), while 2/4 *E. ortleppi* cysts occurred in the lungs and the kidneys each (Table 1).

**Table 1.** Cyst condition and organ location of *Echinococcus* spp. from donkeys in eastern Africa

	*E. granulosus* s. s.	*E. ortleppi*	*E. canadensis* (G6/7)	*E. equinus*
Fertile				
Liver	0	0	3	83
Lungs	7	1	0	28
Total	7	1	3	111
Sterile (viable)				
Liver	1	0	0	0
Lungs	3	0	0	1
Kidney	1	1	0	0
Total	5	1	0	1
Non-viable (caseated/calcified)				
Liver	24	0	12	31
Lungs	33	1	10	20
Kidney	1	1	1	0
Total	58	2	23	51
All conditions				
Liver	25	0	15	115
Lungs	43	2	10	48
Kidney	2	2	1	0
Total	70	4	26	163

**Table 2.** Geographical origin and prevalence of *Echinococcus* spp. from donkeys in eastern Africa

Origin	*N* examined	*N* infected (prevalence)	*N* cysts genotyped	*Echinococcus* spp. (*N* cysts)
1. North/East Kenya, southern Ethiopia				
S-Ethiopia (Borena)	2363	124 (5.2%)	191	EG (23), EC (8), EE (160)
Mandera	75	0 (0.0%)	0	
Marsabit	93	11 (11.8%)^a^	3	EC (3)
Isiolo	42	2 (4.8%)^a^	0	
Garissa	102	8 (7.8%)	3	EO (2), EE (1)
Tana River	32	0 (0.0%)^a^	0	
*Sub-total*	*2707*	*145* (*5.4%)*^a^	*197*	*EG (23), EC (11), EO (2), EE (161)*
2. Northeast Uganda				
Karamoja	70	11 (15.7%)^a^	2	EC (2)
*Sub-total*	*70*	*11* (*15.7%)*^a^	*2*	*EC (2)*
3. Central/West Kenya				
West Pokot	30	0 (0.0%)^a^	0	
Elgeyo Marakwet	102	0 (0.0%)^a^	0	
Baringo	23	0 (0.0%)^a^	0	
Uasin Gishu	14	0 (0.0%)^a^	0	
Laikipia	58	4 (6.9%)^a^	1	EG (1)
Nyandarua	24	0 (0.0%)^a^	0	
Nakuru	93	6 (6.5%)^a^	1	EG (1)
Bomet	419	1 (0.2%)^a^	0	
*Sub-total*	*763*	*11* (*1.4%)*^a^	*2*	*EG (2)*
4. South Kenya/northern Tanzania				
Kitui	95	2 (2.1%)^a^	0	
Machakos	30	0 (0.0%)^a^	0	
Narok	839	17 (2.0%)^a^	6	EG (5), EC (1)
Kajiado/Tanzania	810	13 (1.6%)	3	EG (1), EO (1), EE (1)
Makueni	33	0 (0.0%)	0	
*Sub-total*	*1807*	*32* (*1.8%)*^a^	9	*EG (6), EC (1), EO (1), EE (1)*
**Grand total**^b^	**5961**	**337** (**5.7%)**	**263**	**EG (70), EC (26), EO (4), EE (163)**

EG, *E. granulosus* s. s.; EO, *E. ortleppi*; EC, *E. canadensis* G6/7; EE, *E. equinus*.

a Possible prevalence underestimate, as some positive animals were not included for which the information on the geographical origin was lost during carcass processing.

b Figures include donkeys of unknown origin (within the study region) and positive animals whose data on origin were lost during carcass processing.

**Table 3.** *Echinococcus granulosus* sensu lato representative *nad*1 sequences from donkeys in eastern Africa and details of identical sequences in the GenBank

Isolate	Source	Species	Accession number	Identity sequence accession number	Percentage identity (%)	Length of *nad*1 gene sequence (bp)	Reference	Host	Country
NSD 001	Donkey	*Echinococcus granulosus* sensu stricto	OK489943	MN199128^a^	100	483	Ohiolei *et al*. (2019*a*)	Camel	Nigeria
NSD 024	Donkey	*Echinococcus granulosus* sensu stricto	OK489945	MN199128^a^	99.79	483	Ohiolei *et al*. (2019*a*)	Camel	Nigeria
DMT 077	Donkey	*Echinococcus granulosus* sensu stricto	OK489946	MG672196^a^	100	483	Kinkar *et al*. (2018)	Sheep	Turkey
NSD 006	Donkey	*Echinococcus granulosus* sensu stricto	OK489944	MG672196^a^	99.79	483	Kinkar *et al*. (2018)	Sheep	Turkey
NSD 228	Donkey	*Echinococcus granulosus* sensu stricto	OK489947	MN269987^a^	99.79	894	Ohiolei *et al*. (2019*b*)	Sheep	China
DMT 085	Donkey	*Echinococcus equinus*	OK489948	KP161212	100	483	Wassermann *et al*. (2015)	Zebra	Namibia
NSD 023	Donkey	*Echinococcus ortleppi*	OK489949	KX010904^a^	100	483	Addy *et al*. (2017*a*)	Cattle	Kenya
DMT 086	Donkey	*Echinococcus ortleppi*	OK489950	KX010904^a^	99.79	483	Addy *et al*. (2017*a*)	Cattle	Kenya
NSD 195	Donkey	*Echinococcus ortleppi*	OK489951	KU842044	100	894	Addy *et al*. (2017*a*)	Cattle	Ethiopia
NSD 053	Donkey	*Echinococcus canadensis* (G6/7)	OK489952	MT525967^a^	100	483	Omondi *et al*. (2020)	Camel	Kenya
NSD 256	Donkey	*Echinococcus canadensis* (G6/7)	OK489954	KX010875^a^	100	894	Addy *et al*. (2017*b*)	Camel	Kenya
NSD 033	Donkey	*Spirometra theileri*	OK489955	MN244299	100	332	Eom *et al*. (2019)	Leopard	Tanzania

a Representative reference sequence with closest geographic origin, others with same percentage identity exist.

### Prevalence and geographical distribution of *Echinococcus* spp.

In total, 5961 donkeys were examined, of which 337 (5.7%) were infected with CE and 1 with a metacestode of *S. theileri*. The geographical origin of the donkeys is presented in Table 2 at regional and county level. For 614 donkeys, the origin was unknown from the onset. In addition, from those with known origin, in case of 77 of the 337 positive animals, this information was lost during carcass processing. These positive records were omitted from Table 2 (except for the grand total), resulting for some counties in prevalence underestimates (see footnote ‘a’ in Table 2). Generally, CE prevalence estimates were highest for animals from Karamoja (Uganda), northeastern Kenya and southern Ethiopia, and low for animals from central, western and southern Kenya and northern Tanzania. Geographical structuring was obvious for *E. equinus*, where all except 2 infected animals originated from southern Ethiopia. The other *Echinococcus* spp. were widely spread across the study area; all of the (few) characterized cyst from central Kenya were *E. granulosus* s. s., all cysts from Karamoja were *E. canadensis* (G6/7) (Table 2). The donkey infected with *S. theileri* originated from Kajiado county, southern Kenya.

## Discussion

This study reports for the first time the presence and prevalence of CE in donkeys, and any member of the horse family, in eastern Africa. Data on CE in donkeys are few worldwide, e.g. from Turkey, Italy and Israel (Abo-Shehada, 1988; Mukbel *et al*., 2000; Thompson and McManus, 2002; Oge *et al*., 2004; Varcasia *et al*., 2008; Simsek *et al*., 2015). In Africa, such reports are restricted to the North (Egypt, Tunisia and Morocco), while no data exist from sub-Saharan Africa despite the ubiquity of donkeys in many countries (Pandey, 1980; Azlaf and Dakkak, 2006; Haridy *et al*., 2008; Taha, 2012; Aboelhadid *et al*., 2013; Boufana *et al*., 2014; Lahmar *et al*., 2014; Mahdy *et al*., 2014*a*, 2014*b*; Barghash *et al*., 2017; Desouky *et al*., 2017). The prevalence of donkey CE found in this study (5.7%) was close to that reported from northern Africa with 4.2% in Morocco (Pandey, 1980), 8.5% in Tunisia (Lahmar *et al*., 2014) and 6.9–14.2% in Egypt (Haridy *et al*., 2008; Aboelhadid *et al*., 2013; Mahdy *et al*., 2014*b*; Barghash *et al*., 2017; Desouky *et al*., 2017). In addition to the scarcity of CE data from donkeys, few studies have identified the causative species of *Echinococcus*. Thus, *E. equinus* has been identified in Tunisia and Egypt (Aboelhadid *et al*., 2013; Boufana *et al*., 2014; Lahmar *et al*., 2014; Desouky *et al*., 2017; Mousa *et al*., 2020) and *E. granulosus* s. s. in Tunisia and Morocco (Azlaf, 2007; Boufana *et al*., 2014; Lahmar *et al*., 2014).

*Echinococcus equinus* is assumed to be the species most closely adapted to donkeys and other Equidae as intermediate hosts, reaching high levels of fertility. This was confirmed by 68% fertility among our samples of *E. equinus*. The distribution and host range of this species in sub-Saharan Africa are still rather enigmatic: in our study, almost all donkeys with *E. equinus* originated from southern Ethiopia, only 2 animals from Kenya. This is unexplained, but the scarcity of this species in Kenya is supported by its absence in recent large-scale faecal surveys of dogs (Mulinge *et al*., 2018) and wild carnivores (Kagendo *et al*., 2014) across the country. Older data from Chad (no case of CE in 163 horses and donkeys) suggest that this region of absence of scarcity of *E. equinus* may stretch from eastern to central Africa (Graber *et al*., 1969). No wild equids (zebras) have ever been examined for CE in eastern and central Africa. This is in clear contrast to the situation in southern Africa, where *E. equinus* is obviously common in plains zebras and wild carnivores in northern Namibia (Wassermann *et al*., 2015), was reported from a mountain zebra in ‘South West Africa’ (Kumaratilake *et al*., 1986), and where reports of 60% ‘incidence’ (sic) and fertility of cysts in plains zebra of Kruger National Park in South Africa also suggest the frequent presence of *E. equinus* there (Young, 1975*a*, 1975*b*). The latter is supported by a recent report of fertile cysts of *E. equinus* in a white rhino from Kruger National Park, RSA (Zaffarano *et al*., 2021). There are no reports of *E. equinus* in donkeys, horses or domestic dogs from southern Africa. Although our data close a gap of knowledge by reporting the presence of *E. equinus* in eastern Africa, data on the obviously patchy geographical spread of this species on the continent are still far from complete. In particular, further surveys are needed to investigate whether the endemic area in southern Ethiopia is an isolated focus of this parasite, or is linked to the northern African endemic region. Reasons for differences in local frequency of this parasite are unclear. The principal hosts, donkeys and domestic dogs, are abundant in all parts of our study area. Even though in most parts of eastern Africa, donkeys are rarely slaughtered for human consumption and dogs may not get infected *via* slaughter offal, it is unlikely that carcasses of donkeys that died due to age or accidents are disposed in a way that they are out of reach for (stray) dogs or wild scavengers. Ongoing research on *Echinococcus* transmission in southern Ethiopia may shed light on the risk factors in that focus. This is not irrelevant, as *E. equinus* – long assumed to be apathogenic for humans – has recently been reported as causative agent of human CE (Kim *et al*., 2020; Macin *et al*., 2021), and the rarity of reported human cases may at least partly be due to the general rarity of the parasite in large parts of Africa (and the world).

The numbers of donkeys infected with *Echinococcus* spp. other than *E. equinus* (*E. granulosus* s. s. *n* = 70; *E. canadensis* G6/7 *n* = 26; *E. ortleppi n* = 4) do approximately reflect the relative frequencies of these species in their typical intermediate hosts (sheep, goats, camels and cattle) in the study area. In Kenya, *E. granulosus* s. s. is the most abundant species in sheep, goats and cattle followed by *E. canadensis* (G6/7) and *E. ortleppi*, this frequency can also be seen in donkeys. However, the prevalence of these 3 species in donkeys was far lower than in their ruminant hosts in the same region (Dinkel *et al*., 2004; Maillard *et al*., 2007; Casulli *et al*., 2010; Addy *et al*., 2012; Hailemariam *et al*., 2012; Mutwiri *et al*., 2013; Mbaya *et al*., 2014; Chamai *et al*., 2016; Tigre *et al*., 2016; Odongo *et al*., 2018; Nungari *et al*., 2019; Terefe *et al*., 2019; Tamarozzi *et al*., 2022). Also, cyst fertility rates in donkeys [10% for *E. granulosus* s. s., 12% for *E. canadensis* (G6/7), 1/4 for *E. ortleppi*] were far lower compared to their typical hosts. In case of *E. granulosus* s. s., this is in accordance with studies from the Mediterranean area, where few or no cysts of this species were found fertile in donkeys and horses (Azlaf, 2007; Varcasia *et al*., 2008; Utuk and Simsek, 2013; Boufana *et al*., 2014; Lahmar *et al*., 2014), indicating a poor host adaptation. For *E. canadensis* (G6/7) and *E. ortleppi*, the presented results are even the first confirmation that these species can infect donkeys and reach fertility there; apart from our findings, an *E. ortleppi* cyst of unknown fertility status previously reported from an unspecified species of zebra in Namibia was the only record of *E. ortleppi* from any member of the Equidae (Obwaller *et al*., 2004). Yet, given the low prevalence and fertility rate, donkey infections with *E. granulosus* s. s., *E. canadensis* (G6/7) and *E. ortleppi* are likely the result of spillover from the typical lifecycles between dogs and domestic ruminants, and donkeys probably play a small role, if any, in the transmission of these parasites.

This study reports the unusual finding of *S. theileri* calcified cyst in a donkey that was located in the kidney and initially misidentified as a degenerated *Echinococcus* cyst. Domestic and wild carnivores serve as the definitive hosts of *Spirometra* spp., while the first intermediate hosts are copepods and the second intermediate hosts are amphibians, reptiles or herbivorous mammals (paratenic hosts). A sylvatic cycle for *Spirometra* spp. involving wild herbivores (zebra, warthog and antelope) as second intermediate hosts (paratenic) and carnivores (hyenas) was proposed in Maasai Mara (Nelson *et al*., 1965; Muller-Graf, 1995). A small number of human infections with plerocercoids of *Spirometra* spp. are known from Kenya (Schmid and Watschinger, 1972; MOH, 2016). Although the zoonotic potential of *S. theileri* is unknown, infections with adult worms of this species have been found in leopards and spotted hyenas in Tanzania and recently in domestic dogs in Maasai Mara (Eom *et al*., 2019; Mulinge *et al*., 2021). As the plerocercoid found in our donkey was calcified, the significance of this finding is unclear, and the lifecycle of *S. theileri* in the area is in need of investigation.

## Data Availability

Data supporting results are provided within the article and available on GenBank under accession numbers OK489943-OK489955.
